# Parents Matter: Associations of Parental BMI and Feeding Behaviors With Child BMI in Brazilian Preschool and School-Aged Children

**DOI:** 10.3389/fnut.2018.00069

**Published:** 2018-08-10

**Authors:** Sarah Warkentin, Laís A. Mais, Maria do Rosário Dias de Oliveira Latorre, Susan Carnell, José Augusto de Aguiar Carrazedo Taddei

**Affiliations:** ^1^Discipline of Nutrology, Department of Pediatrics, Federal University of São Paulo, São Paulo, Brazil; ^2^Department of Epidemiology, School of Public Health, University of São Paulo, São Paulo, Brazil; ^3^Division of Child and Adolescent Psychiatry, Department of Psychiatry and Behavioral Sciences, Johns Hopkins University School of Medicine, Baltimore, MD, United States

**Keywords:** child, parents, feeding behavior, obesity, predictors, environment

## Abstract

**Background:** Brazil is undergoing nutritional transition and rates of obesity in preschool and school-aged children are increasing. Excess weight in the first years of life could predict excess weight in adulthood, making it essential to study risk factors in this population.

**Objective:** Our goal was to investigate associations of parent feeding behaviors, as well as more distal familial influences including family SES and maternal and paternal weight, with BMI *z*-score in preschool and school-aged children in a Brazilian sample.

**Methods:** Cross-sectional study. Data were collected in 14 Brazilian private schools. Parents of children aged 2–8 years (*n* = 1,071) completed a questionnaire assessing parent feeding behaviors, as well as sociodemographic and anthropometric information. Hierarchical linear regression models were fitted to investigate relationships between parent and child characteristics and child BMI *z*-score in preschool (2–5 years, *n* = 397) and school-aged (6–8 years, *n* = 618) children.

**Results:** Final models indicated that higher maternal BMI and “restriction for weight control” were associated with higher child BMI *z*-score in both age groups (excessive weight, i.e., BMI ≥ +1 *z*-score, in preschoolers and school-aged children: 24.4 and 35.9%, respectively). In preschoolers only, “healthy eating guidance” and “pressure” were associated with lower child BMI *z*-score. For school-aged children, male sex, higher father BMI, and “restriction for health” were associated with higher child BMI *z*-score.

**Conclusions:** Parent feeding behaviors and parent weight, as well as child sex, are associated with child BMI *z*-score, with evidence for differential relationships in preschool and school-aged children. Optimal obesity prevention and treatment strategies may differ by child age.

## Introduction

The Brazilian population is undergoing nutritional transition, which can be defined as the simultaneous decline of undernourishment and infectious diseases, and rise of overweight, obesity and non-communicable diseases (NCD) (1). From 1996 to 2006, underweight among Brazilian preschoolers decreased 6.3%, while overweight increased 129% (2). The same trend is evident in school-aged children. According to the last national survey in 2008, one in every three children between 5 and 9 years of age had excessive weight. For comparison, prevalence in the mid-70s was one in every 10 children (3). Notably, the vast majority of overweight children globally, amounting to approximately 35 million children, live in developing countries like Brazil (4).

The etiology of obesity is multifactorial (5), involving a variety of biological, economic, social and lifestyle factors (6). Most children now dwell within wider environments rich in availability of various high energy-dense, palatable, cheap, and nutrient-poor foods, where sugary drinks and large portions of food prevail (5, 7, 8). Marketing of less healthy foods is rife at the point of purchase and elsewhere (9). These obesogenic forces in the wider environment are a significant obstacle to healthy eating patterns and body weight in preschool and school-aged children.

Child food intake and expenditure patterns, and thereby child weight, are also influenced by family and community characteristics that may be more distal or more proximal to the child (10). More distally, sociodemographic factors such as parental SES (socioeconomic status) are risk factors for child obesity (11). Notably, although links between lower income and higher BMI *z* are well-established at a between-country level, and within populations of developed countries (12–14), the character of social disparities in obesity within Brazil and other transitioning countries is less clear. For example, consistent with US data, lower parent education was associated with greater child consumption of unhealthy foods in one study (15), but in two others, higher family SES was associated with greater child weight (2, 16).

More proximally, parent (mother and father) weight has been shown to associate with child weight in Brazilian as well as other populations (2, 16, 17). This is likely due to a combination of genetic predisposition and environmental factors (18). For example, body weight is known to be highly heritable in children (19). Genetic and early environment factors as reflected by variables such as maternal pre-pregnancy BMI (Body Mass Index), diabetes and excessive weight gain during pregnancy, prenatal tobacco exposure, high infant birth weight and rapid weight gain during infancy (11), are also known to influence child obesity. Importantly, family environments later in childhood may moderate expression of obesogenic genetic tendencies. For example, a study of children ranging from 1 to 20 years found greater genetic influence on weight among children with less educated parents (20), suggesting environmental moderation of genetic effects to the extent that education was a marker for obesogenic family environment characteristics. In further support of environmental moderation of genetic tendencies, genetic influence on weight is not static but increases throughout childhood (explaining 40% of population variation in BMI *z*-score at 4 years cf. 75% at 19 years), while effects of environmental factors shared by children living in the same home (e.g., aspects of the home food environment) are substantial in younger childhood but diminish with age (20). This likely reflects an increase in expression of genetic risk factors for excess food intake with growing child autonomy over their personal food intake within the home, but also with increasing exposure to the wider obesogenic environment (e.g., opportunities to select food at and after school, outside the home) (20, 21).

Adopting a macro- vs. micro-systems conceptualization (22), parent feeding behaviors may be conceived as more proximally related to children's eating behavior and obesity than sociodemographic and parental anthropometric factors, as well as being more amenable to modification. Importantly, though, relationships with parent feeding strategies may differ depending on the age of the child. Parental behaviors may have a particularly important influence in the preschool years, since parents act as providers, enforcers and role models for young children, who are still highly dependent on them (23–25). In contrast, as children get older, parents may have less direct impacts over their child's food intake, with teachers, peers, and media becoming bigger influences (26). Relationships between parent feeding and child weight are likely bidirectional. For example, while a wealth of cross-sectional studies suggest that restriction is associated with higher child weight and pressure with lower child weight (25), a number of longitudinal studies have supported a model in which parents respond to their perceptions of children's weight and eating behavior rather than vice versa (27–29). However, one recent cohort study found that maternal prompts to eat in children aged 4–5 correlated with child eating in the absence of hunger (EAH) at baseline and 18 months later, while neither child BMI *z*-score nor kcal consumed in the absence of hunger at baseline predicted any of the feeding in feeding strategies at follow up period, supporting mother-to-child influence (30). Notably, reverse relationships between parent feeding and child weight may also differ with age. For example, parents of older children may be more likely to restrict their child's food intake when they perceive him or her to be too heavy, compared to parents who do not have this perception (31).

Since the early years may present the best opportunity to prevent excessive weight gain in Brazil, it is vital to investigate both distal predictors of child weight (e.g., family SES, parent demographic and anthropometric factors) to aid targeting of intervention resources, and proximal predictors (e.g., parent feeding behaviors) to aid development of practical behavioral advice. In light of this, the goal of the current study was to investigate associations of parent feeding behaviors, as well as sociodemographic and anthropometric factors, with child BMI *z*-score, in a large sample of preschool and school-aged children in Brazil. So that we could test the associations of more proximal factors (e.g., parent feeding) without confounding from more distal factors, and establish whether there were independent influences of distal factors that were not mediated by more proximal factors, we used a hierarchical model analysis.

## Materials and methods

### Procedures

This study was part of the *Estudo de Práticas Alimentares* (EPA), a cross-sectional study aiming to adapt and validate the Comprehensive Feeding Practices Questionnaire (CFPQ) for middle- and high-income Brazilian families of preschool (32) and school-aged children (33). Parents of 2-to-8-year-old children, whose children's anthropometric data was complete, were included in this study. We excluded children with diseases that were related to nutrition and/or could influence parental feeding practices; siblings, in order to avoid sample unit duplication, keeping only the youngest child; children who were not in the eligible age group; children from parents who were not born in Brazil; respondents who were not the parent of the index child; parents who completed more than one questionnaire for the same child; and those with missing answers on parental feeding practices questions. To estimate sample size, a type I and a type II probability of 0.05 and 0.20, respectively, were considered. The prevalence of overweight among children was used for this estimation, which resulted in a required sample size of 320 respondents, incorporating over-recruitment to accommodate an anticipated loss of 10% of the original sample. 48 private schools, each serving children ranging in age from 2 to 8 years, in Campinas and São Paulo, Brazil were invited to participate in the study. Of the 16 schools that accepted the invitation, 14 were selected for the current sample, while the other two were selected for piloting. Survey packets containing the questionnaire and instructions requesting the questionnaire's completion within 2 weeks by one of the parents were left in each classroom and distributed to each eligible children. In one school, the questionnaires were administered and completed by parents before a parents–teachers meeting. More details about the procedures are described elsewhere (32, 33). This research received ethical approval from the Federal University of São Paulo (UNIFESP) ethics committee. The mother or the father of each participating child gave written informed consent before completing the survey.

### Measures

Sociodemographic and anthropometric questions included mother and father age, education (middle school complete or incomplete, high-school complete or incomplete, college incomplete or complete), height and weight (parent-report), and family income. Child BMI *z*-score was calculated using child's height, weight, sex and age as reported by the parent, according to the WHO parameters (34). Parental feeding practices were assessed using the Brazilian version of the CFPQ comprising six factors: (1) “Healthy eating guidance” (16 items for preschool children and 15 for school-aged children). This assesses how parents guide their child's eating through encouragement, modeling and teaching about nutrition, e.g., “Do you encourage your child to eat healthy foods before unhealthy ones?” Cronbach's α = 0.83 for both preschool and school-aged children. (2) “Monitoring” (four items for preschool children and six for school-aged children). This assesses how much parents keep track of the unhealthy foods their child consumes, e.g., “How much do you keep track of the sweets (candy, ice cream, cake, pies, and pastries) that your child eats?” Cronbach's α = 0.86 and 0.80 for preschool and school-aged children respectively. (3) “Emotion regulation/food as reward” (six items for preschool and school-aged children). This assesses parents' use of food to regulate their child's emotions and/or as a reward for desirable behaviors, e.g., “When your child gets fussy, is giving him/her something to eat or drink the first thing you do?” Cronbach's α = 0.74 and 0.71 for preschool and school-aged children respectively. (4) “Restriction for weight control” (seven items for preschool and school-aged children). This assesses the degree to which parents restrict their child's food intake to limit or control their child's weight gain, e.g., “I encourage my child to eat less so he/she won't get fat.” Cronbach's α = 0.84 and 0.86 for preschool and school-aged children respectively. (5) “Restriction for health” (five items for preschool and school-aged children). This assesses how much parents restrict their child's food intake to influence their child's health, e.g., “If I did not guide or regulate my child's eating, he/she would eat too much of his/her favorite foods.” Cronbach's α = 0.88 and 0.91 for preschool and school-aged children respectively. (6) “Pressure” (four items for preschool and school-aged children). This assesses how much a parent pressures their child to eat, e.g., “My child should always eat all of the food on his/her plate.” Cronbach's α = 0.72 and 0.76 for preschool and school-aged children respectively). Response options for the CFPQ questions were “never,” “rarely,” “sometimes,” “mostly,” and “always,” or “disagree,” “slightly disagree,” “neutral,” “slightly agree,” and “agree,” on a Likert scale ranging from 1 to 5. The process of transcultural adaptation and validation of the questionnaire is described elsewhere (32, 33). All the CFPQ scores were analyzed as continuous variables, ranging from 1 to 5. All data were double entered with the help of two trained assistant researchers.

### Statistical analysis

**Figure 1:**
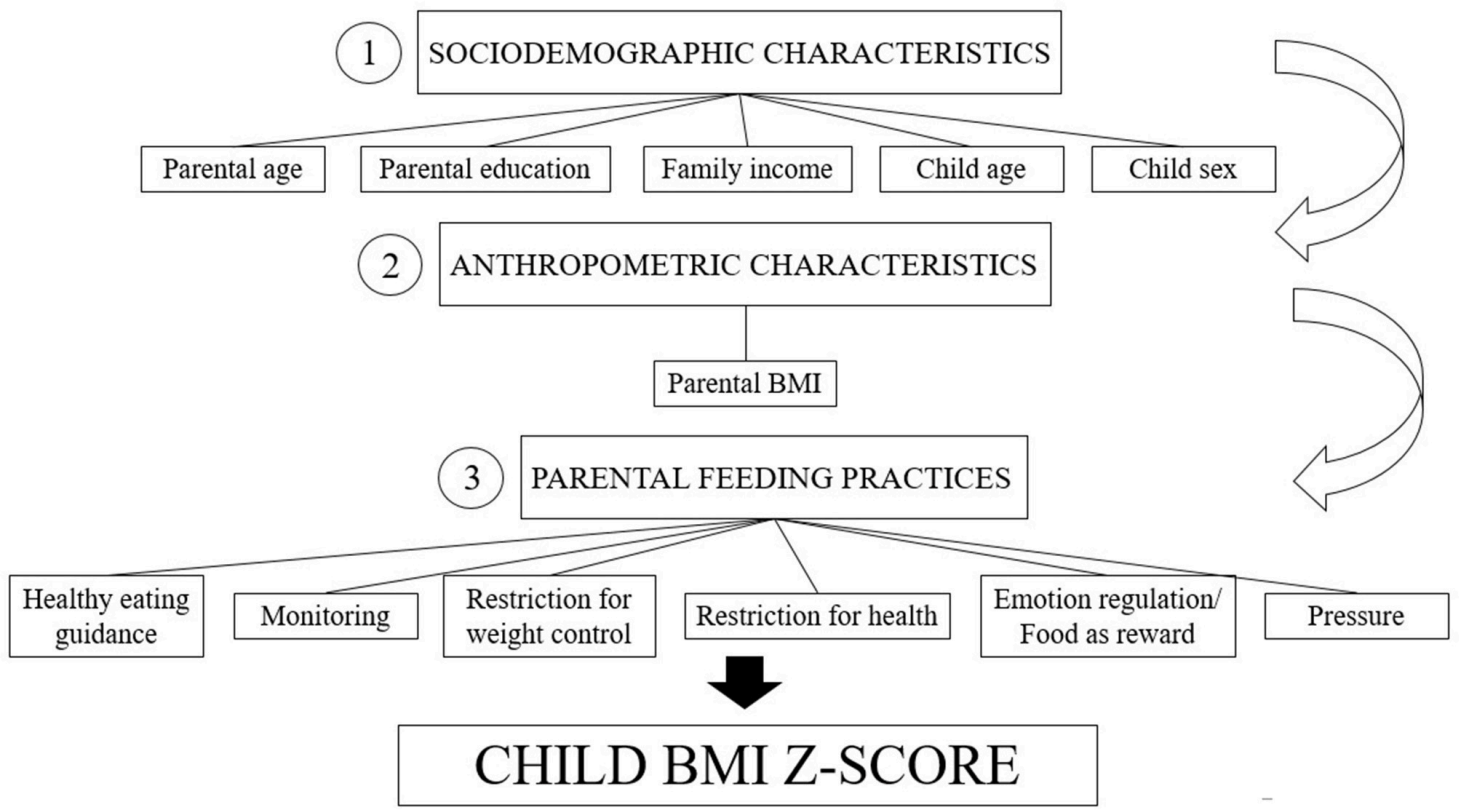
Conceptual hierarchical framework of risk factors for child excessive weight status.

We ran all statistical analyses first for the whole sample and then for preschool and school-aged children separately. We started by running descriptive analyses to explore the data set and to choose appropriate cut-offs for dichotomization of variables of interest. For comparisons between preschool and school-aged children, we ran *t*-tests for continuous variables, and chi-square tests for categorical variables. Most of the independent variables (parental education, family income, child sex) were collected using categorical responses; these were dichotomized for statistical analysis purposes. Parental weight data was ascertained as a continuous variable. Parent and child age and parent feeding practice scores were treated as continuous variables. Since BMI *z*-scores (dependent variable) showed a normal distribution according to the Kolmogorov–Smirnov test, we elected to use linear regression analysis. We then ran a series of simple bivariate linear regressions using the BMI/age *z*-score as the dependent variable, and sociodemographic and anthropometric characteristics and parent feeding practices as independent variables. Multivariate linear regression models for each analysis (whole sample, preschool children, school-aged children) followed recommendations for hierarchical models (22); the hierarchical structure is shown in Figure 1.

Following recommendations by Kleinbaum and Klein (35), variables meeting criteria of *p* ≤ 0.20 in bivariate linear regression analyses were considered eligible for the multivariate analysis at each level. Multivariate analysis was performed first for the variables considered more distal from the child weight status (Model 1: sociodemographic characteristics). This procedure was repeated for anthropometric characteristics (Model 2), and then for the most proximal variables (Model 3: parental feeding practices). Each subsequent model was adjusted for the previous model according to the specified hierarchy, i.e., model 2 was adjusted for model 1, and model 3 was adjusted for models 1 and 2. Variables at each level were entered using the Stepwise Forward entering method, whereby we only included variables with *p* ≤ 0.20, and, further, entered those variables with lowest *p*-values first and those with higher *p*-values at the end, assuming the first variables entered in the model to be the most influential in the equation. This approach represents a balance between a hypothesis-driven and data-driven approach that was designed to create a parsimonious model in which the Model 3 factors (of primary interest) are meaningfully adjusted based on Model 1 and Model 2 factors. Statistical significance was defined as *p* < 0.05. All statistical analyses were performed using the statistical software package Stata version 14.0 (36).

## Results

Table 1 gives descriptive data for sociodemographic and anthropometric characteristics, and parental feeding practices measured by the CFPQ. There were, in total, 984 mothers and 87 fathers, with 410 (38.28%) being parents of preschoolers and 661 (61.72%) parents of school-aged children. Most of the parents had high education and income levels. Mothers of school-aged children were significantly heavier compared to mothers of preschool-aged children. There were no differences in father BMI between age groups.

**Table 1:** Family characteristics for children enrolled in private schools of São Paulo and Campinas, 2014 (*n* = 1,071).

**Independent variables**	**Categories**	**Preschool-aged children (*n* = 410)**	**School-aged children (*n* = 661)**	Whole sample^**^ (*n* = 1,071)	*p*-value^**^
		***n* (%)**	***n* (%)**	***n* (%)**	
**SOCIODEMOGRAPHIC CHARACTERISTICS**					
Mother's age^*^		36.36 (4.55)	38.88 (5.10)	37.91 (5.05)	<**0.001**
Father's age^*^		38.81 (5.81)	41.93 (6.28)	40.73 (6.29)	<**0.001**
Mother's education	Lower than college complete	32 (7.80)	95 (14.37)	127 (11.86)	<**0.05**
	College completed	378 (92.20)	566 (85.63)	944 (88.14)	
Father's education	Lower than college complete	47 (11.60)	134 (20.52)	181 (17.11)	<**0.001**
	College completed	358 (88.40)	519 (79.48)	877 (82.89)	
Family's income	Up to 15 times the minimum wage	149 (38.30)	274 (43.91)	423 (41.76)	>0.05
	More than 15 times the minimum wage	240 (61.70)	350 (56.09)	590 (58.24)	
Child's sex	Male	197 (48.05)	356 (53.86)	553 (51.63)	>0.05
	Female	213 (51.95)	305 (46.14)	518 (48.37)	
**ANTHROPOMETRIC CHARACTERISTICS**					
Mother's BMI^*^		23.22 (3.43)	24.06 (3.82)	23.74 (3.69)	<**0.001**
Father's BMI^*^		26.87 (3.36)	27.06 (3.64)	26.99 (3.54)	>0.05
Child's BMI/age *z*-score^*^		0.21 (1.43)	0.46 (1.36)	0.36 (1.39)	<**0.05**
**PARENTAL FEEDING PRACTICES (CFPQ)**					
Healthy eating guidance^*^		4.38 (0.44)	4.45 (0.42)	4.42 (0.43)	<**0.05**
Monitoring^*^		4.52 (0.70)	4.23 (0.67)	4.34 (0.70)	<**0.001**
Restriction for weight control^*^		2.12 (0.90)	2.24 (0.98)	2.19 (0.95)	<**0.05**
Restriction for health^*^		3.61 (1.14)	3.70 (1.23)	3.66 (1.20)	>0.05
Emotion regulation/Food as reward^*^		1.66 (0.64)	1.39 (0.50)	1.49 (0.57)	<**0.001**
Pressure^*^		3.33 (0.94)	3.30 (1.02)	3.31 (0.99)	>0.05

Brazilian minimum wage in 2014: R$724.00 (US$ 321.77); BMI, body mass index; CFPQ, comprehensive feeding practices questionnaire.

* Continuous variables: means (standard deviation).

** Preschool-aged children vs. school-aged children.

*Values in bold: p ≤ 0.05*.

Table 2 shows results of bivariate analyses for the whole sample, and for the preschool-aged children and school-aged children separately, categorized by each level of the specified hierarchical structure. Bold text indicates statistically significant results (*p* < 0.05).

**Table 2:** Bivariate analyses of associations with child BMI *z*-score for levels 1, 2 and 3 of proposed conceptual hierarchical model, for preschool-aged children, school-aged children and whole sample.

**Variables**	**Risk category**	**Preschool-aged children**				**School-aged children**				**Whole sample**			
		**β**	**SE**	***p*-value**	**CI (95%)**	**β**	**SE**	***p*-value**	**CI (95%)**	**β**	**SE**	***p*-value**	**CI (95%)**
**SOCIODEMOGRAPHIC CHARACTERISTICS**													
Mother's age^*^		−0.02	0.02	>0.05	−0.05; 0.01	0.00	0.10	>0.05	−0.02; 0.02	0.00	0.01	>0.05	−0.02; 0.02
Father's age^*^		0.01	0.01	>0.05	−0.01; 0.04	0.00	0.01	>0.05	−0.02; 0.01	0.00	0.01	>0.05	−0.01; 0.02
Mother's education	Lower than college complete	0.47	0.26	>0.05	−0.04; 0.99	0.24	0.15	>0.05	−0.06; 0.53	**0.34**	0.13	<0.05	0.08; 0.59
Father's education	Lower than college complete	0.32	0.22	>0.05	−0.12; 0.75	**0.28**	0.13	<0.05	0.02; 0.54	**0.32**	0.11	<0.05	0.10; 0.55
Family income	Up to 15 times the minimum wage	0.05	0.15	>0.05	−0.25; 0.34	0.21	0.11	>0.05	0.00; 0.42	0.16	0.09	>0.05	−0.01; 0.34
Child age		0.15	0.09	>0.05	−0.03; 0.32	0.07	0.05	>0.05	−0.02; 0.17	**0.08**	0.02	<0.001	0.04; 0.13
Child sex	Male	−0.01	0.14	>0.05	−0.29; 0.27	**0.26**	0.11	<0.05	0.05; 0.47	**0.17**	0.08	<0.05	0.00; 0.34
**ANTHROPOMETRIC CHARACTERISTICS**													
Mother's BMI^*^		**0.06**	0.02	<0.05	0.02; 0.10	**0.07**	0.01	<0.001	0.05; 0.10	**0.07**	0.01	<0.001	0.05; 0.09
Father's BMI^*^		0.04	0.02	>0.05	−0.01; 0.08	**0.04**	0.01	<0.05	0.01; 0.07	**0.04**	0.01	<0.05	0.02; 0.06
**PARENTAL FEEDING PRACTICES (CFPQ)**													
Healthy eating guidance^*^		−**0.38**	0.16	<0.05	−0.69; −0.06	−0.11	0.13	>0.05	−0.36; 0.14	−0.19	0.10	>0.05	−0.39; 0.00
Monitoring^*^		0.01	0.10	>0.05	−0.19; 0.21	0.05	0.08	>0.05	−0.10; 0.21	0.00	0.06	>0.05	−0.12; 0.12
Restriction for weight control^*^		**0.24**	0.08	<0.05	0.08; 0.40	**0.41**	0.05	<0.001	0.31; 0.52	**0.36**	0.04	<0.001	0.28; 0.45
Restriction for health^*^		**0.14**	0.06	<0.05	0.02; 0.27	**0.18**	0.04	<0.001	0.09; 0.26	**0.17**	0.04	<0.001	0.10; 0.24
Emotion regulation/Food as reward^*^		0.00	0.11	>0.05	−0.22; 0.21	0.07	0.11	>0.05	−0.14; 0.28	−0.02	0.07	>0.05	−0.16; 0.13
Pressure^*^		−**0.23**	0.07	<0.05	−0.38; −0.09	−**0.14**	0.05	<0.05	−0.24; −0.04	−**0.17**	0.04	<0.001	−0.26; −0.09

Brazilian minimum wage in 2014: R$724.00 (US$ 321.77); BMI, body mass index; CFPQ, comprehensive feeding practices questionnaire.

* *Continuous variables*.

*Values in bold: p ≤ 0.05*.

For the whole sample, mother and father education, child age and sex (Model 1), mother and father weight status (Model 2), and “restriction for weight control,” “restriction for health,” and “pressure” (Model 3) were significantly associated with child BMI *z*-score. For the preschool sample, only mother weight status (Model 2), and the parental feeding practices “healthy eating guidance,” “restriction for weight control,” “restriction for health,” and “pressure” (Model 3) were significantly associated with child BMI *z*-score. For the school-aged children, father education and child sex (Model 1), mother and father weight status (Model 2), and “restriction for weight control,” “restriction for health,” and “pressure” (Model 3) were significantly associated with child BMI *z*-score.

Tables 3–**5** shows the results of the multivariate linear regression models for each sample, according to the proposed conceptual hierarchical model. The final model for the whole sample estimated positive associations between child BMI *z*-score and both types of restrictive feeding practices (“restriction for weight control”: β = 0.28, CI 95% 0.19–0.37; “restriction for health”: β = 0.11, CI 95% 0.04–0.18), and a negative association with “pressure” (β = −0.12, CI 95% −0.20 to −0.03). This model was adjusted for father's education, child age, mother's and father's BMI and total variance explained was 10.6% [*F*_(7, 996)_ = 18.06; *R*^2^ adj. = 0.106] (Table 3). The final model for preschool-aged children (Table 4) estimated negative associations between child BMI *z*-score and the parental feeding practices “healthy eating guidance” (β = −0.36, CI 95% −0.67 to −0.04) and “pressure,” (β = −0.22, CI 95% −0.37 to −0.07). In addition, a positive association was found between “restriction for weight control” and child BMI *z*-score (β = 0.24, CI 95% 0.08–0.39). This model was adjusted for mother's BMI and total variance explained was 6.4% [*F*_(4, 392)_ = 7.79; *R*^2^ adj. = 0.064]. The model for school-aged children (Table 5) demonstrated associations between child BMI *z*-score and both “restriction for health” (β = 0.09, CI 95% 0.01–0.18) and “restriction for weight control” (β = 0.36, CI 95% 0.25–0.47) were associated with greater child BMI *z*-score. This model was adjusted for father's education, child sex, both mother's and father's BMI and total variance explained was 13.9% [*F*_(6, 612)_ = 17.57; *R*^2^ adj. = 0.139].

**Table 3:** Final hierarchical model for child BMI *z*-score for whole sample.

**Variables**	**Risk category**	**Model 1**			Model 2^**^			Model 3^***^		
		***F***_**(2, 1055)**_ = **8.74** ***R***^**2**^ **adj**. = **0.014**			***F***_**(4, 1008)**_ = **13.21** ***R***^**2**^ **adj**. = **0.046**			***F***_**(7, 996)**_ = **18.06** ***R***^**2**^ **adj**. = **0.106**		
		**β**	***p*-value**	**CI (95%)**	**β**	***p*-value**	**CI (95%)**	**β**	***p*-value**	**CI (95%)**
**SOCIODEMOGRAPHIC CHARACTERISTICS**										
Mother's age^*^										
Father's age^*^										
Mother's education	Lower than college complete									
Father's education	Lower than college complete	0.27	<**0.05**	**0.04; 0.49**	***0.20***	*>0.05*	*−0.03; 0.43*	*0.16*	*>0.05*	*−0.07; 0.38*
Family income	Up to 15 times the minimum wage									
Child age^*^		0.07	<**0.05**	**0.03; 0.12**	***0.05***	* < 0.05*	*0.00; 0.10*	*0.04*	*>0.05*	*−0.00; 0.09*
Child sex	Male									
**ANTHROPOMETRIC CHARACTERISTICS**										
Mother's BMI^*^					0.06	<**0.001**	**0.04; 0.09**	*0.06*	*<**0.001***	***0.03; 0.08***
Father's BMI^*^					0.03	<**0.05**	**0.00; 0.05**	*0.02*	*>0.05*	*0.00; 0.05*
**PARENTAL FEEDING PRACTICES (CFPQ)**										
Healthy eating guidance^*^										
Monitoring^*^										
Restriction for weight control^*^								0.28	<**0.001**	**0.19; 0.37**
Restriction for health^*^								0.11	<**0.05**	**0.04; 0.18**
Emotion regulation/Food as reward^*^										
Pressure^*^								−0.12	<**0.05**	−**0.20;** −**0.03**

Brazilian minimum wage in 2014: R$724.00 (US$ 321.77); BMI, body mass index; CFPQ, comprehensive feeding practices questionnaire.

* *Continuous variables*.

** *Model adjusted for father's education and child age*.

*** Model adjusted for father's education, child age, mother's BMI and father's BMI.

Values in bold: p ≤ 0.05.

*Level 1: n = 1,058; Level 2: n = 1,013; adjusted for father's education; Level 3: n = 998; adjusted for father's education, child age, mother's and father's BMI*.

*Values in italics: variables used for adjustment of the model*.

**Table 4:** Final hierarchical model for child BMI *z*-score for preschool-aged children.

**Variables**	**Risk category**	**Model 1**			**Model 2**			Model 3^**^		
					***F***_**(1, 403)**_ = **9.17** ***R***^**2**^ **adj**. = **0.020**			***F***_**(4, 392)**_ = **7.79** ***R***^**2**^ **adj**. = **0.064**		
		**β**	***p*-value**	**CI**	**β**	***p*-value**	**CI**	**β**	***p*-value**	**CI**
**SOCIODEMOGRAPHIC CHARACTERISTICS**										
Mother's age^*^										
Father's age^*^										
Mother's education^*^	Lower than college complete									
Father's education^*^	Lower than college complete									
Family income^*^	Up to 15 times the minimum wage									
Child age^*^										
Child sex	Male									
**ANTHROPOMETRIC CHARACTERISTICS**										
Mother's BMI^*^					0.06	<**0.05**	**0.02; 0.10**	***0.05***	*<0.05*	*0.00; 0.09*
Father's BMI^*^										
**PARENTAL FEEDING PRACTICES (CFPQ)**										
Healthy eating guidance^*^								−0.36	<**0.05**	−**0.67;** −**0.04**
Monitoring^*^										
Restriction for weight control^*^								0.24	<**0.05**	**0.08; 0.39**
Restriction for health^*^										
Emotion regulation/Food as reward^*^										
Pressure^*^								−0.22	<**0.05**	−**0.37; 0.07**

Brazilian minimum wage in 2014: R$724.00 (US$ 321.77); BMI, body mass index; CFPQ, comprehensive feeding practices questionnaire.

* Continuous variables.

** *Model adjusted for mother's BMI*.

Values in bold: p ≤ 0.05.

*Level 2: n = 405; Level 3: n = 397; adjusted for mother's BMI*.

*Values in italics: variables used for adjustment of the model*.

**Table 5:** Final hierarchical model for child BMI *z*-score for school-aged children.

**Variables**	**Risk category**	**Model 1**			Model 2^**^			Model 3^***^		
		***F***_**(2, 650)**_ = **5.10** ***R***^**2**^ **adj**. = **0.012**			***F***_**(4, 618)**_ = **9.89** ***R***^**2**^ **adj**. = **0.054**			***F***_**(6, 612)**_ = **17.57** ***R***^**2**^ **adj**. = **0.139**		
		**β**	***p*-value**	**CI**	**β**	***p*-value**	**CI**	**β**	***p*-value**	**CI**
**SOCIODEMOGRAPHIC CHARACTERISTICS**										
Mother's age^*^										
Father's age^*^										
Mother's education	Lower than college complete									
Father's education	Lower than college complete	0.27	<**0.05**	**0.01; 0.53**	*0.15*	*>0.05*	*−0.12; 0.42*	*0.12*	*>0.05*	*−0.14; 0.38*
Family income	Up to 15 times the minimum wage									
Child age^*^										
Child sex	Male	0.25	<**0.05**	**0.04; 0.46**	*0.28*	*<0.05*	*0.07; 0.49*	*0.31*	*<0.05*	*0.11; 0.52*
**ANTHROPOMETRIC CHARACTERISTICS**										
Mother's BMI^*^					0.07	<**0.001**	**0.04; 0.10**	***0.06***	*<0.001*	*0.04; 0.09*
Father's BMI^*^					0.03	<**0.05**	**0.00; 0.06**	***0.02***	*>0.05*	*0.00; 0.05*
**PARENTAL FEEDING PRACTICES (CFPQ)**										
Healthy eating guidance^*^										
Monitoring^*^										
Restriction for weight control^*^								0.36	<**0.001**	**0.25; 0.47**
Restriction for health^*^								0.09	<**0.05**	**0.01; 0.18**
Emotion regulation/Food as reward^*^										
Pressure^*^										

Brazilian minimum wage in 2014: R$724.00 (US$ 321.77); BMI, body mass index; CFPQ, comprehensive feeding practices questionnaire.

* Continuous variables.

** *Model adjusted for father's education and child sex*.

*** Model adjusted for father's education, child sex, mother's BMI and father's BMI.

Values in bold: p ≤ 0.05.

*Level 1: n = 653. Level 2: n = 623; adjusted for father's education. Level 3: n = 618; adjusted for father's education, child sex, both mother's and father's BMI*.

*Values in italics: variables used for adjustment of the model*.

## Discussion

Using hierarchical models capturing three different layers of influence varying in proximity to child outcomes, we determined sociodemographic, anthropometric and behavioral factors associated with child BMI *z*-score in a large sample of preschool- and school-aged children in Brazil. The following discussion of results is divided into the applied three levels of analysis, progressing from more distal to more proximal factors.

### Level 1—sociodemographic characteristics

Parental educational level is an indicator of family SES and has been repeatedly linked to greater risk of being overweight, in both preschool- and school-aged children [e.g., Bammann et al. (37)]. Notably, in the current study this association was only found in the Model 1 of the hierarchical models, not remaining statistically significant in the final models. Importantly, this suggests that effects of education on child BMI *z*-score may be mediated by factors more proximal to child outcomes including parental BMI and the parent feeding behaviors remaining in the final models. The final models also demonstrated that among the older children (school-aged), boys had higher BMI *z*-scores than girls. Since BMI *z*-scores already adjust for differential BMI trajectories by sex based on international reference data, this finding could reflect the presence of greater obesity risk for school-aged boys than girls in this specific Brazilian sample.

### Level 2—parent's anthropometric characteristics

Confirming previous studies (17, 37, 38), greater weight in both mothers and fathers was significantly associated with higher child BMI *z*-score. Notably, this association was present even after controlling for education, which was weakly associated with maternal BMI (*r* = 0.12, *p* = 0.0001), and included in Level 1 (data not shown). Notably, whereas preschoolers showed an increase of 0.06 in BMI *z*-score for every point of mother's BMI, this increment was similar but greater (β = 0.07) in the school-aged sample. This could result from greater cumulative exposure of the child to obesogenic environmental influences conferred by an overweight mother with time, but could also reflect increases in genetic and potentially epigenetic influences on weight through development. For example, a longitudinal twin study conducted in the UK found that genetic influences on weight status become increasingly stronger from 4 to 11 years, potentially because when children get older they become more independent and are able to exert more control over what they eat, and this enables them to act in accordance with their genetic propensity (39). Notably, although maternal overweight has most frequently been linked to child's weight [e.g., Costa Ribeiro et al. (18)], here we also observed an influence of father weight, among school-aged children only (β = 0.03, CI 95% 0.00–0.06). This suggests that lifestyle behaviors of the whole family could contribute to obesity risk at this age, and is also consistent with a genetic explanation such that genetic risk conferred by both parents is increasingly expressed with age. Nevertheless, within a family system, each family member shapes and is shaped by the other family members' actions, influencing mutually different patterns (40), and fathers, although neglected in much research thus far, play an increasingly large role on child feeding (41). Our findings support consideration of anthropometric status of both parents in attempts to develop effective family-based behavioral interventions (42).

### Level 3—parental feeding practices

Parental feeding practices are thought to influence child weight via effects on eating behavior (43). However, a dominant line of thinking suggests that certain parent feeding practices may have opposite effects to the parent's goals (44). For example, recent studies found that parental restriction of preschoolers' food intake with the goal of controlling their weight was associated with a risk of 1.75 for child excessive weight (17) as well as increased intake of foods high in fat and sugar (45). Potentially, restricting young child's food intake may be useful in the short-term, lowering the intake of sweets and high fat foods, but may backfire as the child gets older, increasing their preference for the palatable foods that were previously restricted (45). Consistent with this hypothesis, in the current study we observed positive associations between restriction and child BMI *z*-score in both preschool and school-aged children, with a larger effect among the older children (β = 0.37 i.e., an increase of 0.37 BMI *z*-score points with each point of the restriction score vs. β = 0.20 i.e., a 0.20 BMI *z*-score difference) and greater total variance explained was 13.9% [*F*_(6, 612)_ = 17.57; *R*^2^ adj. = 0.139]. However, strong experimental or interventional evidence to support a causal relationship between parent restriction and excessive weight gain in children is lacking, and longitudinal (28, 46) and mediational (47) analyses are more supportive of a model in which parents react to greater appetite and weight in children by attempts to restrict intake. Our results may therefore be more reflective of this “child-response” explanation.

“Pressure to eat” may be defined as parents' insistence or demands that their child eat more food, using strategies like the insisting that the child cleans the plate, or compelling the child to eat one or two more bites (44). Research has consistently found that pressuring is associated with lower child weight (29, 48, 49). However, this finding should not be interpreted as indicating protection against child overweight—rather, parents who perceive their child as too thin, adopt a pressuring strategy with the goal of increasing child weight (44). In the current study, we observed a robust relationship between parental pressure and lower weight, which only remained significant in the final model for the preschooler sample (β = −0.22) and whole sample (β = −0.12). This could reflect a greater impact of pressure in younger children, who are in the very early stages of developing eating habits and therefore more likely to demonstrate both food neophobia and selectivity (49). Alternatively, this developmental pattern of behavior may be more likely to elicit parental pressure.

The “Healthy eating guidance” factor of the adapted version of the CFPQ that we used captures a variety of structuring practices, such as providing a healthy food environment, teaching about nutrition, and modeling healthy eating habits (32, 33). Strategies such as teaching the child about nutrition and providing fruits and vegetables at home have been consistently associated with preschoolers' consumption of healthier foods (50, 51), suggesting protection against excessive weight gain. Consistent with this previous research, we found associations with lower BMI *z*-score, but only for the preschool sample (β = −0.36, CI 95% −0.67 to −0.04). Interestingly, scores on this factor were also significantly, although only incrementally, higher in the school-aged group. This suggests that although parents may be marginally less inclined to adopt structural strategies to promote healthy eating at preschool age, they may have relatively strong impacts on child weight, supporting encouragement of these strategies at this developmental stage. A related implication is that structuring strategies are of less value for obesity protection at older child ages, supporting differential targets of intervention in later childhood.

Our study had some limitations. First, the cross-sectional design does not allow causal inference. However, longitudinal studies of other cohorts have demonstrated that relationships between parental feeding practices and child weight are likely bidirectional (27–29, 48, 52), supporting a bidirectional interpretation of our own results. Second, child and parent anthropometric data were self-reported by the parent; this may have introduced inaccuracies and bias. Although the use of self-report is a practical way to assess specific information in large-scale studies like this, we are aware of the biases of its use. However, about 70% of the anthropometric information provided by parents was derived from pediatrician/medical reports or measured at home (data not shown), and parents of children of this age group are likely to be more aware of child's anthropometrics due to contact with health professionals for checks on development (53). Additionally, parental perceptions may be a particularly salient measure to assess when studying subjective processes such as feeding practices (51). Third, the sample of 87 fathers was relatively small, limiting possible conclusions about the influence on child weight status. Further testing of paternal influences in samples with a larger number of fathers is required to confirm our findings. Fourth, in our sample the education level of the parents was homogenously high, potentially limiting the education effects that we were able to detect, and preventing generalizability to lesser educated populations.

Another potential limitation is that factors at all hierarchical levels differed between our preschool and school-aged groups. Older parent age for the older children is to be expected. Greater parent education levels for the younger sample were not predicted but the difference, while significant in this large sample, was small in scale. Since the preschool and school-aged samples were drawn from the same schools, facilitating comparison across the groups, these differences between age groups are likely meaningful and generalizable to other populations. Further, when we repeated analyses of age group differences in parent feeding practice scores adjusting for Level 1 and 2 sociodemographic and anthropometric characteristics (family income, mother and father education, mother and father age) as well as child BMI *z*-score, all values remained significant, except for restriction for weight control. Follow-up analyses adjusting for each Level 1 and 2 factor separately revealed that the age difference in restriction for weight control became non-significant with control for child BMI *z*-score. This does not compromise our findings but instead aids interpretation by suggesting that the slightly larger association between this score and child BMI *z*-score that we saw for the older children may partly be driven by the greater variation within the upper end of the BMI distribution in older children. We therefore do not believe the observed differences between age groups compromise the meaning and generalizability of the potential age group differences in patterns of predictors that we revealed.

Strengths of our design include the previous translation and validation of the CFPQ in a Brazilian sample of preschool and school-aged children, which provides confidence that the parental feeding practices assessed here were appropriate for the sample, facilitating generalization of the findings to populations with the same characteristics. The use of a large sample maximized our power to detect reliable and meaningful associations and generate accurate estimates of associative strength. Finally, our hierarchical approach was a suitable, powerful, and controlled strategy for investigating potential determinants of a complex phenomenon such as child weight.

To conclude, the results of this large study in a Brazilian cohort of parents using a hierarchical analysis to evaluate the associations between parental feeding behaviors, parental weight and child sex with child BMI *z*-score suggested that certain associations may differ in presence/absence or in strength according to child's age. For example, final models indicated that maternal weight and the use of “restriction for weight control” were associated with higher child weight in both age groups but the effect was slightly stronger in older children. The use of positive feeding practices and “pressure” were associated with lower child weight only for younger children, while male sex and paternal weight were associated with higher child weight in older children, with a smaller effect in the same direction for the use of “restriction for health.” These findings suggest that positive parent feeding practices may be helpful for obesity protection particularly in younger children, pressure to eat may be unhelpful or even counterproductive in younger children, restriction may be unhelpful or even counterproductive in both age groups, and mother weight might be considered an indication for intervention for younger children while both mother and father weight might be considered indications among older children. More generally, our results support age-appropriate targeting and tailoring of intervention approaches by health professionals and others for optimal prevention of childhood overweight.

## Ethics statement

This research received ethical approval from the Federal University of São Paulo (UNIFESP) ethics committee. All participants gave written informed consent before completing the survey.

## Author contributions

SW contributed to the study design, participated in the data gathering and entering, performed the data analysis and interpretation, and wrote the article. LM contributed to the study design, participated in the data gathering and entering, performed the data analysis and interpretation, and wrote the article. ML suggested and supervised the data analysis and interpretation, and reviewed the article. SC contributed to conceptualization and writing of the article. JT selected the study design, supervised the data gathering and data entering, and reviewed the article. All authors approved the contents of the manuscript.

### Conflict of interest statement

The authors declare that the research was conducted in the absence of any commercial or financial relationships that could be construed as a potential conflict of interest.
